# Comparison of Cumulative Live Birth Rates Between GnRH-A and PPOS in Low-Prognosis Patients According to POSEIDON Criteria: A Cohort Study

**DOI:** 10.3389/fendo.2021.644456

**Published:** 2021-06-21

**Authors:** Shaodi Zhang, Yisha Yin, Qiuyuan Li, Cuilian Zhang

**Affiliations:** ^1^ Reproductive Medicine Center, Henan Provincial People’s Hospital, Zhengzhou, China; ^2^ People’s Hospital of Henan University, People’s Hospital of Zhengzhou University, Zhengzhou, China

**Keywords:** GnRH antagonist, progestin-primed ovarian stimulation (PPOS), *in vitro* fertilization (IVF), cumulative live birth rate, Patient Oriented Strategies Encompassing Individualized Oocyte Number (POSEIDON)

## Abstract

**Objective:**

To compare the cumulative live birth rate (CLBR) of a gonadotropin-releasing hormone (GnRH) antagonist regimen and a progestin-primed ovarian stimulation (PPOS) regimen in low-prognosis patients according to POSEIDON criteria.

**Design:**

Single-center, retrospective, observational study.

**Setting:**

Henan Provincial People’s Hospital, Zhengzhou, China

**Patients:**

Women aged ≤40 years, with a body mass index <25 kg/m^2^, who underwent *in vitro* fertilization (IVF) or intracytoplasmic sperm microinjection (ICSI) and met POSEIDON low-prognosis criteria.

**Intervention:**

GnRH or PPOS regimen with IVF or ICSI.

**Main Outcome Measure:**

CLBR per oocyte retrieval cycle.

**Results:**

Per oocyte retrieval cycle, CLBR was significantly higher with GnRH antagonist versus PPOS (35.3% vs 25.2%; P<0.001). In multivariable logistic regression analysis, CLBR per oocyte retrieval cycle was significantly lower with PPOS versus GnRH antagonist before (OR 0.62 [95% confidence intervals (CI): 0.46, 0.82; P=0.009]) and after (OR 0.66 [95% CI: 0.47, 0.93; P=0.0172]) adjustment for age, body mass index, infertility type, infertility duration, baseline follicle stimulating hormone, anti-Müllerian hormone (AMH), antral follicle count (AFC), and insemination method. CLBR was numerically higher with the GnRH antagonist regimen than with PPOS, across all of the POSEIDON groups, and was significantly higher in patients aged ≥35 years with poor ovarian reserve [AFC <5, AMH <1.2 ng/mL] (unadjusted, P=0.0108; adjusted, P=0.0243).

**Conclusion:**

In this single-center, retrospective, cohort study, patients had a higher CLBR with a GnRH antagonist versus PPOS regimen, regardless of other attributes.

## Introduction

Poor ovarian response (POR) affects between 9% and 24% (1, 2) of women undergoing assisted reproduction and is characterized by a failure to respond adequately to standard protocols and to recruit adequate follicles, resulting in reduced oocyte production and a diminished probability of pregnancy (3). POR is a therapeutic challenge that is amplified by a lack of consensus on the definition of POR and on the appropriate therapeutic approach for women with previous POR (4).

The introduction of the Bologna criteria (5) in 2011 attempted to standardize the definition of POR, although subsequent research suggested a number of critical issues that prevented widespread acceptance (6, 7), including a lack of adequate patient stratification (8). Indeed, published data have indicated that pregnancy outcomes evaluated using the Bologna criteria are widely variable according to the patient subgroup selected for analysis (9–11). More recently, the Patient-Oriented Strategies Encompassing Individualized Oocyte Number (POSEIDON) standard was proposed to assist with the identification and management of POR (12). The POSEIDON criteria stratify women by age, ovarian biomarkers, and ovarian response to previous stimulatory treatments (12, 13), and better characterize women with diminished ovarian reserves and those with POR compared with the Bologna criteria (8, 12). As a result, patients undergoing assisted reproduction, who have expected or unexpected impaired ovarian response can now be stratified into four clear and distinct subgroups (12, 13), aiding both clinicians and researchers to formulate more optimal management plans.

In terms of treatment for women with POR, gonadotropin-releasing hormone (GnRH) analogs for pituitary suppression are currently used in routine practice, and antagonistic GnRH analogs, administered *via* subcutaneous injection, are the most frequently used regimens (14). The effectiveness and safety profiles of the GnRH antagonists allow for a flexible treatment approach across a wide spectrum of women requiring assisted reproduction, including those with POR (15).

Progestin-primed ovarian stimulation (PPOS) is another commonly used regimen in patients with POR. However, the benefits of the PPOS regimen (including the lower cost, oral administration, and reduced risk of ovarian hyperstimulation syndrome vs GnRH regimens) are countered by the need for ‘freeze-all’ cycles and the inability to pursue a more rapid fresh embryo transfer procedure (16, 17).

Evidence to date suggests that the PPOS regimen may provide similar or better clinical outcomes compared with conventional regimens in patients with POR (17–20); however, data comparing long-term outcomes of GnRH antagonist and PPOS regimens (such as cumulative live birth rate [CLBR]) according to POSEIDON groups are lacking. Recently, the GnRH regimen for the treatment of women with POR has been increasing in popularity, with evidence suggesting that it achieves better clinical outcomes compared with unconventional regimens such as microstimulation (21). This raises the question as to whether the GnRH regimen can achieve better long-term clinical outcomes compared with the PPOS regimen, another unconventional regimen, in the POR setting. Thus, in the present study, we compared the CLBR with a GnRH antagonist regimen and PPOS regimen in low-prognosis patients according to the POSEIDON criteria, with the aim of providing insights to help guide treatment decisions.

## Methods

### Study Design

This was a single-center, retrospective, cohort, observational study of women who underwent assisted reproductive technology (ART) at the Reproductive Medicine Center of Henan Provincial People’s Hospital (Zhengzhou) between January 2016 and December 2018.

The study protocol was approved by the medical ethics committees of Zhengzhou University and Henan Provincial People’s Hospital, and complied with the Declaration of Helsinki. The data were anonymous, and the requirement for informed consent was therefore waived.

### Patients

Women aged ≤40 years with a body mass index (BMI) <25 kg/m^2^ who underwent *in vitro* fertilization (IVF) or intracytoplasmic sperm microinjection (ICSI) procedures and met low-prognosis POSEIDON criteria were included in the study. The POSEIDON criteria were applied to determine the prognostic group of each eligible woman (8): group 1: age <35 years, normal ovarian reserve (antral follicle count [AFC] ≥5, anti-Müllerian hormone [AMH] ≥1.2 ng/mL), ≤9 oocytes retrieved after standard ovarian stimulation in the previous cycle; group 2: age ≥35 years, normal ovarian reserve (AFC ≥5, AMH ≥1.2 ng/mL), ≤9 oocytes retrieved after standard ovarian stimulation in the previous cycle; group 3: age <35 years, poor ovarian reserve (AFC <5, AMH <1.2 ng/mL); and group 4: age ≥35 years, poor ovarian reserve (AFC <5, AMH <1.2 ng/mL). All women were required to have completed all embryo transfers or have had a live birth by 20 June 2020 from a single IVF oocyte retrieval cycle performed between 2016 and 2018.

Exclusion criteria included: 1) uterine endometrial polyps, uterine adhesions, or abnormal uterine anatomical structure; 2) endocrine disorders such as abnormal thyroid function and hyperprolactinemia; 3) tuberculosis of reproductive system and other systemic diseases; 4) women who received preimplantation genetic screening or preimplantation genetic diagnosis, and those with known chromosomal abnormalities; and 5) frozen oocytes or oocytes obtained *via* donation. At the end of follow-up, women who had no live birth reported but who had an ongoing clinical pregnancy or had embryos remaining were excluded.

### Clinical Setting

The ART used in the study consisted of either a GnRH antagonist regimen or a PPOS regimen. In the GnRH antagonist regimen, ovarian stimulation was initiated from day 2–3 of menstruation with intramuscular injections of human menopausal gonadotropin (HMG; Lobode, Livzon Group Livzon Pharmaceutical Co., Ltd.) or follicle stimulating hormone (FSH; Lishenbao, Livzon Group Livzon Pharmaceutical Co., Ltd.) at a dose of 150–300 IU/day until hCG trigger day. The dose of gonadotropin was adjusted during the stimulation process according to follicular development, which was determined by ultrasound and serum hormone levels, up to the maximum of 300 IU/day. A daily dose of 0.25 mg GnRH antagonist was initiated when a dominant follicle reached a mean diameter of 12 mm or when blood luteinizing hormone (LH) levels began to show a notable upward trend; the dose was continued until the day of hCG administration.

In the PPOS regimen, patients received oral medroxyprogesterone acetate (Xian Iu, Zhejiang Xianju Pharmaceutical Co., Ltd.) 10 mg/day and HMG (LoBode, Livzon Pharmaceutical Co., Ltd.) or urinary FSH (Lishenbao, Livzon Pharmaceutical Co., Ltd.) intramuscular injection at 150–300 IU/day from day 2–3 of menstruation until hCG trigger day. As per the GnRH regimen, the dosage of gonadotropin was adjusted according to the follicular response.

For both regimens, an hCG trigger injection (Lishenbao, Livzon Pharmaceutical Co., Ltd.) was administered at a dose of 8000–10000 IU. Patients with five or fewer dominant follicles received a dose of 10000 IU; all other patients received a dose of 8000 IU. Trigger day occurred when three follicles of ≥16 mm diameter, two follicles of ≥17 mm diameter, or one follicle of ≥18 mm diameter were observed.

### Embryo Transfer

Vaginal ultrasound-guided oocyte retrieval was performed 33–36 h after trigger injection. Depending on male semen parameters, routine IVF (normal parameters) or ICSI microinjection (abnormal parameters) was performed. Embryos were incubated for 3–5 days prior to selection, and either transferred or frozen.

For patients who had undergone the GnRH antagonist regimen, if the endometrium was in good condition (thickness ≥8 mm; acceptable morphology) and there were no contraindications for transfer, a fresh cycle transfer could be performed. Alternatively, viable embryos were vitrified and frozen, and patients underwent elective frozen embryo transfer. For patients who underwent PPOS treatment, all embryos were frozen.

For patients receiving freeze-thaw embryos and who had a regular menstrual cycle, the endometrium was monitored *via* vaginal ultrasound, progesterone was administered on the day of ovulation, and the cleavage-stage embryos or blastocysts were respectively transferred 3 or 5 days after ovulation. For patients receiving freeze-thaw embryos and who had irregular menstruation, oral estradiol valerate supplementation (1 mg) was administered on days 2–4 of the menstrual cycle, followed by a flexible dose up to day 11–20 (4 mg/day to a maximum of 8 mg/day) according to endometrial thickness. When intima thickness was ≥8 mm, the dose of estradiol valerate was fixed, and progesterone administered. Cleavage-stage embryos or blastocysts were respectively transferred on days 4 or 6 after progesterone administration.

Luteal support was initiated on the day of oocyte retrieval in a fresh embryo cycle or from the start of endometrium transformation in a frozen embryo transfer. Support consisted of oral dydrogesterone tablets (Duphaston Helansuwei Pharmaceutical company) 10 mg twice daily and 8% progesterone sustained-release vaginal gel (Xenoto, Merck Serono, Germany). After embryo transfer, the estrogen and progesterone doses were kept unchanged until the blood β-hCG was checked 14 days after transfer. If pregnancy continued to support the corpus luteum, the estrogen and progesterone doses were gradually tapered from week 8 and then discontinued by week 10.

### Outcomes

The primary outcome of the study was CLBR per oocyte retrieval cycle, defined as the probability of a live birth from an ovarian stimulation, including all fresh and frozen embryo transfers from that stimulation. Neonates over 28 weeks of gestation with one of four vital signs (heartbeat, breathing, umbilical cord pulsation, and voluntary muscle contraction) after delivery were considered live births, and the period of delivery of live births was defined as the live birth cycle. Multiple births in a single pregnancy were considered a single live birth. Cumulative live births per oocyte retrieval cycle were the first live births obtained from all embryos obtained after one oocyte retrieval.

### Data Collection

Demographic, clinical, and laboratory data were obtained from the hospital database. Variables of clinical and laboratory indicators included total gonadotropin (Gn), total Gn days and Gn starting dose, hCG day estradiol dose, LH level, progestin level, and endometrial thickness. In addition, the numbers of dominant follicles (≥14 mm), oocytes obtained, mature oocytes (metaphase II), normal fertilizations (two pronuclei), and available embryos (i.e., embryos meeting the standards of transfer or freezing), and the clinical pregnancy rate were recorded, along with the number of stimulation cycles without oocytes, the rate of unharvested oocytes in the retrieval cycle, and the cycle cancellation rate. The clinical pregnancy rate was defined as the gestational period/the number of transfer cycles × 100%. Adjustment variables (baseline indicators) included age, BMI, AFC, AMH, basic FSH, years of infertility, type of infertility (primary, secondary), and final insemination method (IVF/ICSI).

### Statistical Analysis

All normally distributed and skewed continuous variables were expressed as mean (standard deviation) or median (interquartile range). Categorical variables were expressed as frequencies (%). Variables between groups were compared using independent sample t test and chi-square test. The Kruskal–Wallis test was applied for the variables with a skewed distribution.

Logistic regression was used to compare the effects of two ovulation induction schemes (GnRH antagonist and PPOS) on the CLBR per oocyte retrieval cycle. Crude regression estimates are presented, as well as estimates adjusted for baseline covariates. We selected confounders on the basis of their associations with the outcomes of interest or a change in effect estimate of more than 10% (22).

To examine the robustness of our results, we conducted interaction and stratified analyses according to the POSEIDON criteria, age (<35 and ≥35 years), insemination method (IVF/ICSI), BMI (15.20–20.32 kg/m^2^, 20.40–22.00 kg/m^2^ and 22.10–24.98 kg/m^2^), type of fertility (primary/secondary), duration of infertility (<2, ≥2 to <5, and ≥5 years), baseline FSH (1.84–7.08 IU/L, 7.09–9.67 IU/L and 9.70–29.43 IU/L), AMH (<1.2 ng/mL and ≥1.2 ng/mL), and AFC (<5 and ≥5).

To ensure that our results were not biased by the inclusion of various controlled ovarian stimulation (COS) cycles in the same women or by the transfer time due to the number of available embryos, we performed four sensitivity analyses; in the first, we used multiple imputation, based on five replications and a chained equation approach to account for missing data; in the second, we restricted the analysis to the first COS cycle, ensuring each patient was only included once; in the third, we restricted the analysis to the COS cycle where 1–3 embryos were available; and finally, in the fourth, to generate a matched population between the two groups, we used a greedy 1:1 matching algorithm. We used calipers of 0.05 on the propensity score scale and 1:1 sampling without replacement. P<0.05 was considered statistically significant. All statistical analyses were performed using Empower Stats (www.empowerstats.com, X&Y solutions, Inc. Boston MA) and R software version 3.4.3 (http://www.r-project.org).

## Results

### Patient Disposition

Out of 4110 cycles using GnRH antagonist or PPOS regimens between January 2016 and December 2018, 1024 IVF/ICSI cycles met the inclusion criteria (**Supplementary Figure 1**). As of the follow-up date, it was not possible to determine whether a cumulative live birth had been achieved (embryos remaining) in 104/1024 cycles, including 33 cycles in the GnRH antagonist group and 71 cycles in the PPOS group. In total, 920 cycles (GnRH antagonist regimen, n=459; PPOS regimen, n=461) were included in the analysis.

#### Patient Demographics and Characteristics

Patient demographics and characteristics were generally similar between the groups, with differences only seen in baseline FSH (P=0.002) and AMH (P=0.019; **Table 1**). When comparing the clinical and laboratory indicators between the two COS regimens, the GnRH antagonist regimen was associated with significantly lower median LH on hCG trigger day, greater mean endometrial thickness, and higher median number of dominant follicles, number of oocytes retrieved, number of mature oocytes, number of normal fertilized oocytes, and number of usable embryos on day 3 (all P-values <0.05; **Table 2**). The rate of unretrieved oocytes in the oocyte retrieval cycle was 1.5% and 2.6% for the GnRH antagonist and PPOS regimens, respectively (P=0.250).

**Table 1:** Characteristics of the two COS regimen groups.

Parameters	GnRH antagonist regimen	PPOS regimen	*P*-value
No. of cycles	459	461	
No. of patients	426	409	
Age, years	33.6 ± 4.7	33.8 ± 4.6	0.662
<35	107 (23.3)	106 (23.0)	
≥35	226 (49.2)	231 (50.1)	
Body mass index, kg/m^2^	21.3 ± 1.7	21.1 ± 1.8	0.243
T1 (15.20–20.32)	140 (30.5)	154 (33.4)	
T2 (20.40–22.00)	154 (33.6)	157 (34.1)	
T3 (22.10–24.98)	165 (36.0)	150 (32.5)	
Type of infertility	n=459	n=461	0.696
Secondary infertility	282 (61.4)	289 (62.7)	
Primary infertility	177 (38.6)	172 (37.3)	
Duration of infertility, years	n=459 3 (2, 6)	n=460 3 (2, 6)	0.955
T1 (<2)	111 (24.2)	105 (22.8)	
T2 (≥2, <5)	189 (41.2)	189 (41.1)	
T3 (≥5)	159 (34.6)	166 (36.1)	
Baseline FSH (U/L)	n=388 8.16 (6.45, 10.45)	n=368 8.19 (6.76, 11.68)	0.002
T1 (1.84–7.08)	139 (35.8)	112 (30.4)	
T2 (7.09–9.67)	127 (32.7)	126 (34.2)	
T3 (9.70–29.43)	122 (31.4)	130 (35.3)	
AMH (ng/mL)	n=395 1.21 (0.60, 2.44)	n=385 0.92 (0.51, 2.14)	0.019
<1.2	196 (49.62)	223 (57.92)	
≥1.2	199 (50.38)	162 (42.08)	
AFC (n)	n=447 6 (4, 9)	n=437 5 (3, 8)	0.075
<5	156 (34.9)	189 (43.3)	
≥5	291 (65.1)	248 (56.8)	
Insemination method	n=452	n=449	0.775
IVF	331 (73.2)	325 (72.4)	
ICSI	121 (26.8)	124 (27.6)	
POSEIDON group	n=459	n=461	0.175
1	72 (15.7)	76 (16.5)	
2	147 (32.0)	129 (28.0)	
3	79 (17.2)	104 (22.6)	
4	161 (35.1)	152 (33.0)	

Data presented as mean ± standard deviation, median (Q1, Q3), or number and percentage of cycles, n (%).

Missing data: the number of years of infertility was missing in one case, baseline FSH was missing in 162 cases, AMH was missing in 138 cases, and AFC was missing in 36 cases.

AFC, antral follicle count; AMH, anti-Müllerian hormone; COS, controlled ovarian stimulation; FSH, follicle stimulating hormone; GnRH, gonadotropin-releasing hormone; ICSI, intra-cytoplasmic sperm injection; IVF, in vitro fertilization; PPOS, progestin-primed ovarian stimulation; T, trisection.

**Table 2:** Comparison of clinical and laboratory indicators between two COS regimens.

Parameters	GnRH antagonist regimen	PPOS regimen	*P*-value
N	459	461	
Total dosage of Gn used (IU)	1799.10 ± 676.67	1819.69 ± 741.01	0.661
Duration of Gn use (days)	7 (6, 9)	7 (6, 9)	0.340
Starting dose of Gn (IU)	228.97 ± 55.08	228.63 ± 43.59	0.916
On hCG injection day			
Estradiol (pg/mL)	744.00 (456.80, 1048.00)	738.70 (443.45, 1143.75)	0.176
LH (U/L)	2.49 (1.52, 4.20)	3.50 (2.22, 5.34)	0.010
Progesterone (ng/mL)	0.46 (0.29, 0.71)	0.48 (0.32, 0.69)	0.449
Endometrial thickness (mm)	9.11 ± 2.60	6.63 ± 2.05	<0.001
Number of dominant follicles	3 (2, 5)	3 (2, 4)	0.027
Number of oocytes retrieved	4 (3, 6)	3 (2, 5)	0.001
Number of mature oocytes	4 (2, 5)	3 (2, 5)	0.002
Number of normal fertilized oocytes	2 (1,4)	2 (1, 3)	0.017
Number of useable embryos on day 3	2 (1, 3)	2 (1, 3)	0.002
Cycle cancellation rate due to a lack of useable embryos	64 (13.9)	82 (17.8)	0.111
Number of stimulation cycles without oocytes	7 (1.5)	12 (2.6)	0.250
Cumulative pregnancy rate per oocyte retrieval cycle	215 (46.8)	162 (35.1)	<0.001
Cumulative live birth rate per oocyte retrieval cycle	162 (35.3)	116 (25.2)	<0.001

Data presented as mean ± standard deviation, median (Q1, Q3), or number and percentage of cycles, n (%). The mean ± standard deviation of the D3 available embryo number was 2.35 ± 1.89 in the GnRH antagonist group and 1.98 ± 1.65 in the PPOS group.

COS, controlled ovarian stimulation; Gn, gonadotropin; GnRH, gonadotropin-releasing hormone; hCG, human chorionic gonadotropin; LH, luteinizing hormone; PPOS, progestin-primed ovarian stimulation.

#### Cumulative Live Birth Rate

The CLBR per oocyte retrieval was statistically significantly higher with the GnRH antagonist regimen versus the PPOS regimen (35.3% vs 25.2%; P<0.001; **Table 2**). A univariate logistic regression model was used to study the factors affecting the clinical outcome of CLBR per oocyte retrieval cycle. COS regimen, age, baseline FSH, AMH, and AFC were shown to be influencing factors (**Supplementary Table 1**). Multivariable logistic regression analysis showed that CLBR per oocyte retrieval cycle was significantly lower with PPOS vs the GnRH antagonist regimen both before (odds ratio [OR] 0.62 [95% CI: 0.46, 0.82; P=0.009]) and after (OR 0.66 [95% CI: 0.47, 0.93; P=0.0172]) adjustment for age, BMI, infertility type, duration of infertility, baseline FSH, AMH, AFC and insemination method (**Table 3**). Stratification was performed, taking into account potential confounding effects. After stratification, the effects of the regimens on CLBR were in the same direction (**Supplementary Table 2**).

**Table 3:** Multivariable logistic regression analysis results of the two COS regimen groups and clinical outcomes.

COS regimen	Cumulative live birth rate per oocyte retrieval cycle			
	Before adjustment		After adjustment	
	OR (95% CI)	*P*-value	OR (95% CI)	*P*-value
GnRH antagonist regimen	0.62 (0.46, 0.82)	0.0009	0.66 (0.47, 0.93)	0.0172
PPOS regimen				

Adjustments were made for age, body mass index, infertility type, duration of infertility, baseline FSH, AMH, AFC and insemination method. Multiple imputation, based on five replications and a chained equation approach, was used to account for missing data. Analyses performed on original data excluded any period during which the adjusted variable was missing. Analyses performed with imputed data were performed on the total population (no missing data).

AFC, antral follicle count; AMH, anti-Müllerian hormone; CI, confidence interval; COS, controlled ovarian stimulation; FSH, follicle stimulating hormone; GnRH, gonadotropin-releasing hormone; OR, odds ratio; PPOS, progestin-primed ovarian stimulation.

Multivariate logistic regression analysis of CLBR per oocyte retrieval cycle stratified by the four POSEIDON groups showed that CLBR was numerically higher with the GnRH antagonist regimen than with PPOS, regardless of the POSEIDON group (**Table 4**), although the possible influence of the small sample sizes must be considered. No significant differences in CLBR were observed between treatments in POSEIDON groups 1–3. The CLBR was significantly lower in patients in group 4 (age ≥35 years, poor ovarian reserve [AFC <5, AMH <1.2 ng/mL]) who underwent PPOS versus the GnRH antagonist regimen (unadjusted, P=0.0108; adjusted, P=0.0243).

**Table 4:** Multivariate logistic regression analysis results of the cumulative live birth rate per oocyte retrieval cycle by COS regimen and Poseidon group.

POSEIDON group	COS regimen	Statistics	Cumulative live birth rate per oocyte retrieval cycle			
			Before adjustment		After adjustment	
			OR (95% CI)	*P*-value	OR (95% CI)	*P*-value
1	GnRH antagonist	31.9% (23/72)	0.66 (0.32, 1.36)	0.2631	0.83 (0.34, 2.04)	0.6829
	PPOS	23.7% (18/76)				
2	GnRH antagonist	32.0% (47/147)	0.62 (0.36, 1.06)	0.0794	0.95 (0.46, 1.96)	0.8963
	PPOS	22.5% (29/129)				
3	GnRH antagonist	29.1% (23/79)	0.77 (0.40, 1.49)	0.4400	0.59 (0.23, 1.53)	0.2808
	PPOS	24.0% (25/104)				
4	GnRH antagonist	42.9% (69/161)	0.54 (0.34, 0.87)	0.0108	0.53 (0.30, 0.92)	0.0243
	PPOS	29.0% (44/152)				

Adjustments were made for: age, body mass index, infertility type, duration of infertility, baseline FSH, AMH, AFC and insemination method. Multiple imputation, based on five replications and a chained equation approach, was used to account for missing data. Analyses performed on original data excluded any period during which the adjusted variable was missing. Analyses performed with imputed data were performed on the total population (no missing data).

AFC, antral follicle count; AMH, anti-Müllerian hormone; CI, confidence interval; COS, controlled ovarian stimulation; FSH, follicle stimulating hormone; GnRH, gonadotropin-releasing hormone; OR, odds ratio; PPOS, progestin-primed ovarian stimulation.

The robustness of these findings was assessed in sensitivity analyses, and the results were confirmed.

## Discussion

To our knowledge, this is the first study to examine the impact of regimen choice (GnRH antagonist versus PPOS) on CLBR in low-prognosis patients according to POSEIDON groups. In this single-center, retrospective, cohort study, use of a GnRH antagonist regimen provided significantly higher CLBR compared with PPOS, before and after adjustment for patient factors (age, BMI, infertility type, duration of infertility, baseline FSH, AMH, AFC, and insemination method) in POSEIDON patients. In addition, after stratification by POSEIDON group, a numerically higher CLBR in groups 1–3 and a significantly higher CLBR in group 4 was observed with the GnRH antagonist regimen versus the PPOS regimen. Thus, our study demonstrates that regimen choice is an influencing factor on CLBR, and that the conventional GnRH antagonist regimen may have advantages over the PPOS regimen among patients with low prognosis according to POSEIDON criteria.

PPOS can be used for the treatment of women with POR, and published evidence has suggested that PPOS may provide similar or better clinical outcomes compared with conventional regimens in patients with POR (17–20). However, patient heterogeneity and suboptimal study design have hampered interventional clinical trials in POR (8). This has led to conflicting results and a lack of evidence-based guidance to support effective intervention in this patient population (8).

Several studies have retrospectively analyzed POR patients according to Bologna criteria and found that the PPOS regimen could effectively inhibit premature LH surge and resulted in higher rates of metaphase II oocyte, fertilization, good-quality embryos, and live births compared with the GnRH regimen (23–25). Another recent study found no difference in CLBR in POR patients according to the Bologna criteria, irrespective of the type of pituitary suppression (26). Compared with the Bologna criteria, the POSEIDON criteria provide a more detailed stratification of low-prognosis patients (12). The international POSEIDON Group (8, 12) and Chinese Embryology Expert Group (27) have recommended that POSEIDON patients may benefit from receiving conventional regimens; however, to date, there has been a lack of data comparing the efficacy of different regimens in this population. Thus, the results of our study, which support the use of a GnRH antagonist regimen, rather than a PPOS regimen, in all patients with POR are both timely and of clinical relevance for physicians and patients making ART treatment decisions.

Reproductive outcomes of ART treatment are traditionally reported as pregnancies per cycle or per embryo transfer; however, CLBR gives a more long-term view of the chance of ART success. In our study, CLBR was numerically higher with the GnRH antagonist regimen than with PPOS, across all of the POSEIDON groups. Importantly, these outcomes remained observable even after stratification for factors such as age, BMI, infertility type and duration, baseline hormone levels, and insemination procedure. Prior studies have shown that CLBR generally decreases with increasing POSEIDON group (across several different regimens) (28, 29). In our analysis, numerical differences in CLBR were observed between treatments in POSEIDON groups 1–3, and the GnRH regimen was statistically superior to PPOS in patients in POSEIDON group 4. Although the POSEIDON standard provides the possibility for different types of patients with low prognosis to follow individualized ovulation induction programs, our findings indicate that all patients with POR, regardless of other attributes, may gain more benefit from a GnRH antagonist regimen than from a PPOS regimen.

Several factors may account for the higher CLBR in the GnRH antagonist regimen group compared with the PPOS regimen group. In particular, significantly more oocytes were retrieved among women receiving the GnRH regimen (4 vs 3, P=0.001) and two key indicators of oocyte quality (number of mature oocytes and number of normal fertilizations) were elevated in the GHRH-treated patients, all of which are prognostic factors for live birth rate (30–33). In addition, although advances in cryopreservation mean that frozen embryo transfer is considered to be almost as effective as fresh transfer (34), concerns remain that embryonal damage caused during the freeze-thaw process may contribute to reduced viability (35, 36). We can speculate that this also may have contributed to the lower CLBR associated with the PPOS regimen.

In our study, we found that the cycle cancellation rate was lower in women who received the GnRH antagonist regimen than those who received the PPOS regimen. While this finding is in line with previous reports of studies conducted in women with normal ovarian response (37) or those with polycystic ovarian syndrome (38), it contradicts the findings of other studies. For example, a study published by Huang et al. (24) reported a lower cycle cancellation rate in women who received the PPOS regimen versus those who received the GnRH antagonist regimen. However, although their study investigated a population of women with poor ovarian response, they did not apply the POSEIDON criteria. We speculate that differences in cycle cancellation rates among studies may be attributable to differences in the characteristics and sizes of the patient populations in each of these studies. We also reported a higher number of mature oocytes with the GnRH antagonist regimen than with the PPOS regimen. A recently published meta-analysis reported that more oocytes were obtained with the GnRH antagonist regimen compared with PPOS regimens (39), which we speculate may mean that there was a higher number of mature oocytes with the GnHR antagonist regimen.

Our study had several advantages. The sample size of 4110 cycles was larger than many previous studies and our findings were robust due to the use of multivariate logistic regression and stratification analysis, with results confirmed *via* sensitivity analyses. There were also several notable limitations. As a retrospective, single-center study, our results need to be further evaluated in future randomized, controlled clinical trials with a larger population. Furthermore, only women aged ≤40 years with a BMI <25 kg/m^2^ who met POSEIDON low-prognosis criteria participated in our study, so we cannot draw conclusions about other population groups. These inclusion criteria were applied because female obesity and advanced age have been shown to impair IVF outcome (40–42). Importantly, a recent meta-analysis reported that a BMI >30 kg/m^2^ was significantly associated with fewer live births compared with a normal BMI (18–24.9 kg/m^2^) (43). To avoid the impact of obesity on outcomes, we excluded women with a BMI ≥25 mg/m^2^. However, we acknowledge that this may have introduced bias into our study. Regarding the PPOS regimen in this study, oral medroxyprogesterone acetate was used in the IVF center in which this study was conducted. It is unknown whether our results can be extrapolated to other types of progestin, such as dydrogesterone or dienogest. Finally, in the present study, we did not make conservative or optimal estimates of CLBR. Conservative estimates of CLBR assume that patients who do not return for treatment have no chance of achieving an ART-related live birth, whereas optimal estimates assume that women discontinuing treatment would have the same chance of achieving a live birth as those continuing treatment. In this study, women who had remaining embryos but had not yet achieved a live birth at the end of the follow-up period were excluded. Thus, the conclusions from this study cannot be extrapolated to the entire spectrum of ART patients with POR seen in the clinic.

## Conclusion

In this single-center, retrospective, cohort study, a significantly higher CLBR was reported with a GnRH antagonist regimen compared with a PPOS regimen among all ART patients with low prognosis according to POSEIDON criteria, especially those aged ≥35 years with poor ovarian reserve.

## Data Availability Statement

The original contributions presented in the study are included in the article/**Supplementary Material**. Further inquiries can be directed to the corresponding author.

## Ethics Statement

The studies involving human participants were reviewed and approved by Zhengzhou University and Henan Provincial People’s Hospital. Written informed consent for participation was not required for this study in accordance with the national legislation and the institutional requirements.

## Author Contributions

SZ was responsible for research conception and data collection. YY and QL were responsible for data processing and interpretation. CZ was responsible for providing data and guiding research. All authors contributed to the article and approved the submitted version.

## Funding

The development of this publication was financially supported by Merck Serono Co. Ltd. China, an affiliate of Merck KGaA, Darmstadt, Germany. The authors declare that this study received funding from Merck Serono Co. The funder was not involved in the study design, collection, analysis, interpretation of data, the writing of this article or the decision to submit it for publication.

## Disclaimer

The views and opinions described in this publication do not necessarily reflect those of the grantor.

## Conflict of Interest

The authors declare that the research was conducted in the absence of any commercial or financial relationships that could be construed as a potential conflict of interest.

## References

[1] Keay SD Liversedge NH Mathur RS Jenkins JM. Assisted Conception Following Poor Ovarian Response to Gonadotrophin Stimulation. Br J Obstet Gynaecol (1997) 104:521–7. 10.1111/j.1471-0528.1997.tb11525.x 9166190

[2] Ubaldi F Vaiarelli A D’Anna R Rienzi L. Management of Poor Responders in IVF: Is There Anything New? BioMed Res Int (2014) 2014:352098. 10.1155/2014/352098 25136579 PMC4127291

[3] Abu-Musa A Haahr T Humaidan P. Novel Physiology and Definition of Poor Ovarian Response; Clinical Recommendations. Int J Mol Sci (2020) 21:2110. 10.3390/ijms21062110 PMC7139860 32204404

[4] Patrizio P Vaiarelli A Levi Setti PE Tobler KJ Shoham G Leong M. How to Define, Diagnose and Treat Poor Responders? Responses From a Worldwide Survey of IVF Clinics. Reprod BioMed Online (2015) 30:581–92. 10.1016/j.rbmo.2015.03.002 25892496

[5] Ferraretti AP La Marca A Fauser BC Tarlatzis B Nargund G Gianaroli L. ESHRE Consensus on the Definition of ‘Poor Response’ to Ovarian Stimulation for In Vitro Fertilization: The Bologna Criteria. Hum Reprod (2011) 26:1616–24. 10.1093/humrep/der092 21505041

[6] Ferraretti AP Gianaroli L. The Bologna Criteria for the Definition of Poor Ovarian Responders: Is There a Need for Revision? Hum Reprod (2014) 29:1842–5. 10.1093/humrep/deu139 25008235

[7] Younis JS Ben-Ami M Ben-Shlomo I. The Bologna Criteria for Poor Ovarian Response: A Contemporary Critical Appraisal. J Ovarian Res (2015) 8:76. 10.1186/s13048-015-0204-9 26577149 PMC4650906

[8] Esteves SC Roque M Bedoschi GM Conforti A Humaidan P Alviggi C. Defining Low Prognosis Patients Undergoing Assisted Reproductive Technology: POSEIDON Criteria - The Why. Front Endocrinol (Lausanne) (2018) 9:461. 10.3389/fendo.2018.00461 30174650 PMC6107695

[9] Bozdag G Polat M Yarali I Yarali H. Live Birth Rates in Various Subgroups of Poor Ovarian Responders Fulfilling the Bologna Criteria. Reprod BioMed Online (2017) 34:639–44. 10.1016/j.rbmo.2017.03.009 28366519

[10] Busnelli A Papaleo E Del Prato D La Vecchia I Iachini E Paffoni A. A Retrospective Evaluation of Prognosis and Cost-Effectiveness of IVF in Poor Responders According to the Bologna Criteria. Hum Reprod (2015) 30:315–22. 10.1093/humrep/deu319 25432927

[11] La Marca A Grisendi V Giulini S Sighinolfi G Tirelli A Argento C. Live Birth Rates in the Different Combinations of the Bologna Criteria Poor Ovarian Responders: A Validation Study. J Assist Reprod Genet (2015) 32:931–7. 10.1007/s10815-015-0476-4 PMC4491085 25925345

[12] Poseidon Group (Patient-Oriented Strategies Encompassing IndividualizeD Oocyte Number) Alviggi C Andersen CY Buehler K Conforti A De Placido G. A New More Detailed Stratification of Low Responders to Ovarian Stimulation: From a Poor Ovarian Response to a Low Prognosis Concept. Fertil Steril (2016) 105:1452–3. 10.1016/j.fertnstert.2016.02.005 26921622

[13] Humaidan P Alviggi C Fischer R Esteves SC. The Novel POSEIDON Stratification of ‘Low Prognosis Patients in Assisted Reproductive Technology’ and its Proposed Marker of Successful Outcome. F1000Res (2016) 5:2911. 10.12688/f1000research.10382.1 28232864 PMC5302217

[14] Wang Y Kuang Y Chen Q Cai R. Gonadotropin-Releasing Hormone Antagonist Versus Progestin for the Prevention of Premature Luteinising Hormone Surges in Poor Responders Undergoing *In Vitro* Fertilisation Treatment: Study Protocol for a Randomised Controlled Trial. Trials (2018) 19:455. 10.1186/s13063-018-2850-x 30134964 PMC6106816

[15] Copperman AB Benadiva C. Optimal Usage of the GnRH Antagonists: A Review of the Literature. Reprod Biol Endocrinol (2013) 11:20. 10.1186/1477-7827-11-20 23496864 PMC3618003

[16] La Marca A Capuzzo M. Use of Progestins to Inhibit Spontaneous Ovulation During Ovarian Stimulation: The Beginning of a New Era? Reprod BioMed Online (2019) 39:321–31. 10.1016/j.rbmo.2019.03.212 31138494

[17] Massin N. New Stimulation Regimens: Endogenous and Exogenous Progesterone Use to Block the LH Surge During Ovarian Stimulation for IVF. Hum Reprod Update (2017) 23:211–20. 10.1093/humupd/dmw047 28062551

[18] Chen Q Wang Y Sun L Zhang S Chai W Hong Q. Controlled Ovulation of the Dominant Follicle Using Progestin in Minimal Stimulation in Poor Responders. Reprod Biol Endocrinol (2017) 15:71. 10.1186/s12958-017-0291-0 28870217 PMC5583982

[19] Wang L Yin M Liu Y Chen Q Wang Y Ai A. Effect of Frozen Embryo Transfer and Progestin-Primed Ovary Stimulation on IVF Outcomes in Women With High Body Mass Index. Sci Rep (2017) 7:7447. 10.1038/s41598-017-07773-w 28785018 PMC5547067

[20] Wang Y Chen Q Wang N Chen H Lyu Q Kuang Y. Controlled Ovarian Stimulation Using Medroxyprogesterone Acetate and hMG in Patients With Polycystic Ovary Syndrome Treated for IVF: A Double-Blind Randomized Crossover Clinical Trial. Med (Baltimore) (2016) 95:e2939. 10.1097/MD.0000000000002939 PMC4782886 26945402

[21] Lijun S Bei Z Jijun H Yingying F Junwei Z Zhe L. Application of Three Kinds of Ovulation Induction in In Vitro Fertilization/Intracytoplasmic Sperm Injection Patients With Poor Ovarian Response. Chin J Reprod Contracept (2018) 3. [In Chinese].

[22] Jaddoe VW de Jonge LL Hofman A Franco OH Steegers EA Gaillard R. First Trimester Fetal Growth Restriction and Cardiovascular Risk Factors in School Age Children: Population Based Cohort Study. BMJ (2014) 348:g14. 10.1136/bmj.g14 24458585 PMC3901421

[23] Mu Z Sa Y Sun Z Yi Y. Ovulation Induction With High Progesterone Levels may be More Suitable for Elderly Patients With Low Ovarian Response. J Gynecol Obstet Hum Reprod (2019) 50:101661. 10.1016/j.jogoh.2019.101661 31809957

[24] Huang P Tang M Qin A. Progestin-Primed Ovarian Stimulation is a Feasible Method for Poor Ovarian Responders Undergoing in IVF/ICSI Compared to a GnRH Antagonist Protocol: A Retrospective Study. J Gynecol Obstet Hum Reprod (2019) 48:99–102. 10.1016/j.jogoh.2018.10.008 30321608

[25] Huang MC Tzeng SL Lee CI Chen HH Huang CC Lee TH. GnRH Agonist Long Protocol Versus GnRH Antagonist Protocol for Various Aged Patients With Diminished Ovarian Reserve: A Retrospective Study. PloS One (2018) 13:e0207081. 10.1371/journal.pone.0207081 30403766 PMC6221355

[26] Chen Q Chai W Wang Y Cai R Zhang S Lu X. Progestin vs. Gonadotropin-Releasing Hormone Antagonist for the Prevention of Premature Luteinizing Hormone Surges in Poor Responders Undergoing In Vitro Fertilization Treatment: A Randomized Controlled Trial. Front Endocrinol (Lausanne) (2019) 10:796. 10.3389/fendo.2019.00796 31824419 PMC6882854

[27] Chinese Expert Suggestions Group. Suggestions of Chinese Experts on the Diagnosis and Treatment of Low-Prognosis Patients Undergoing Assisted Reproductive Technology: A Delphi Analysis. In Chinese. Chin J Reprod Contracep (2020) 40:353–60.

[28] Shi W Zhou H Tian L Zhao Z Zhang W Shi J. Cumulative Live Birth Rates of Good and Low Prognosis Patients According to POSEIDON Criteria: A Single Center Analysis of 18,455 Treatment Cycles. Front Endocrinol (Lausanne) (2019) 10:409. 10.3389/fendo.2019.00409 31293519 PMC6606694

[29] Li Y Li X Yang X Cai S Lu G Lin G. Cumulative Live Birth Rates in Low Prognosis Patients According to the POSEIDON Criteria: An Analysis of 26,697 Cycles of In Vitro Fertilization/Intracytoplasmic Sperm Injection. Front Endocrinol (Lausanne) (2019) 10:642. 10.3389/fendo.2019.00642 31608011 PMC6761219

[30] Sunkara SK Rittenberg V Raine-Fenning N Bhattacharya S Zamora J Coomarasamy A. Association Between the Number of Eggs and Live Birth in IVF Treatment: An Analysis of 400 135 Treatment Cycles. Hum Reprod (2011) 26:1768–74. 10.1093/humrep/der106 21558332

[31] Steward RG Lan L Shah AA Yeh JS Price TM Goldfarb JM. Oocyte Number as a Predictor for Ovarian Hyperstimulation Syndrome and Live Birth: An Analysis of 256,381 In Vitro Fertilization Cycles. Fertil Steril (2014) 101:967–73. 10.1016/j.fertnstert.2013.12.026 24462057

[32] Zhao Z Shi H Li J Zhang Y Chen C Guo Y. Cumulative Live Birth Rates According to the Number of Oocytes Retrieved Following the “Freeze-All” Strategy. Reprod Biol Endocrinol (2020) 18:14. 10.1186/s12958-020-00574-3 32087702 PMC7035702

[33] Hariton E Kim K Mumford SL Palmor M Bortoletto P Cardozo ER. Total Number of Oocytes and Zygotes are Predictive of Live Birth Pregnancy in Fresh Donor Oocyte In Vitro Fertilization Cycles. Fertil Steril (2017) 108:262–8. 10.1016/j.fertnstert.2017.05.021 PMC5545054 28601410

[34] Bosch E De Vos M Humaidan P. The Future of Cryopreservation in Assisted Reproductive Technologies. Front Endocrinol (Lausanne) (2020) 11:67. 10.3389/fendo.2020.00067 32153506 PMC7044122

[35] Argyle CE Harper JC Davies MC. Oocyte Cryopreservation: Where are We Now? Hum Reprod Update (2016) 22:440–9. 10.1093/humupd/dmw007 27006004

[36] De Santis L Nottola SA Coticchio G Borini A Iussig B Miglietta S. Type of Protein Supplement in Cryopreservation Solutions Impacts on the Degree of Ultrastructural Damage in Frozen-Thawed Human Oocytes. Cryobiology (2020) 95:143–50. 10.1016/j.cryobiol.2020.03.010 32243889

[37] Iwami N Kawamata M Ozawa N Tamamoto T Watanabe E Moriwaka O. New Trial of Progestin-Primed Ovarian Stimulation Using Dydrogesterone Versus a Typical GnRH Antagonist Regimen in Assisted Reproductive Technology. Arch Gynecol Obstet (2018) 298:663–71. 10.1007/s00404-018-4856-8 30069600

[38] Eftekhar M Hoseini M Saeed L. Progesterone-Primed Ovarian Stimulation in Polycystic Ovarian Syndrome: An Rct. Int J Reprod BioMed (2019) 17:671–6. 10.18502/ijrm.v17i9.5103 PMC6804324 31646262

[39] Ata B Capuzzo M Turkgeldi E Yildiz S La Marca A. Progestins for Pituitary Suppression During Ovarian Stimulation for ART: A Comprehensive and Systematic Review Including Meta-Analyses. Hum Reprod Update (2021) 27:48–66. 10.1093/humupd/dmaa040 33016316

[40] Bellver J Ayllon Y Ferrando M Melo M Goyri E Pellicer A. Female Obesity Impairs In Vitro Fertilization Outcome Without Affecting Embryo Quality. Fertil Steril (2010) 93:447–54. 10.1016/j.fertnstert.2008.12.032 19171335

[41] Yan J Wu K Tang R Ding L Chen ZJ. Effect of Maternal Age on the Outcomes of *In Vitro* Fertilization and Embryo Transfer (IVF-ET). Sci China Life Sci (2012) 55:694–8. 10.1007/s11427-012-4357-0 22932885

[42] Semondade N Huberlant S Bourhis-Lefebrvre V Arbo E Gallot V Colombani M. Female Obesity is Negatively Associated With Live Birth Rate Following IVF: A Systematic Review and Meta-Analysis. Hum Reprod Update (2019) 25:439–51. 10.1093/humupd/dmz011 30941397

[43] Bu Z Dai W Guo Y Su Y Zhai J Sun Y. Overweight and Obesity Adversely Affect Outcomes of Assisted Reproductive Technologies in Polycystic Ovary Syndrome Patients. Int J Clin Exp Med (2013) 6:991–5. PMC3832340 24260609

