# Supplementary material for: Comparison of Cumulative Live Birth Rates Between GnRH-A and PPOS in Low-Prognosis Patients According to POSEIDON Criteria: A Cohort Study
Source: Front Endocrinol (Lausanne). 2021 Jun 21;12:644456. doi: 10.3389/fendo.2021.644456 (PMC8256850; doi:10.3389/fendo.2021.644456)
Supplement: Supplementary file 1 [file DataSheet_1.docx]

Supplementary Material

# Supplementary Tables

**Supplementary Table 1.** Single factor analysis of the factors affecting clinical outcomes

| Parameter | Statistics  (n=901) | Cumulative live birth rate per oocyte retrieval cycle | |  |
| --- | --- | --- | --- | --- |
|  |  | OR (95%CI) | *P*-value |  |
| Ovulation stimulation program |  |  |  |  |
| GnRH antagonist regimen | 459 (49.9) | 0.62 (0.46, 0.82) | 0.0009 |  |
| PPOS regimen | 461 (50.1) |  |  |  |
| Age, years | 33.7 ± 4.7 | 0.94 (0.91, 0.97) | <0.0001 |  |
| Insemination method |  |  |  |  |
| IVF | 656 (72.8) | 0.93 (0.68, 1.29) | 0.6742 |  |
| ICSI | 245 (27.2) |  |  |  |
| Body mass index, kg/m^2^ | 21.19 ± 1.77 | 1.00 (0.92, 1.08) | 0.9979 |  |
| Type of infertility |  |  |  |  |
| Secondary | 571 (62.1) | 1.18 (0.88, 1.57) | 0.2647 |  |
| Primary | 349 (37.9) |  |  |  |
| Duration of infertility, years | 3 (2, 6) | 0.96 (0.92, 1.01) | 0.0850 |  |
| Baseline FSH, U/L | 8.18 (6.61, 10.91) | 0.95 (0.91, 0.98) | 0.0071 |  |
| AMH, ng/mL | 1.08 (0.56, 2.30) | 1.08 (1.03, 1.14) | 0.0039 |  |
| AFC, n | 6 (3, 9) | 1.04 (1.01, 1.07) | 0.0111 |  |

Data presented as mean ± standard deviation, median (Q1, Q3), or number and percentage of cycles, n (%).

AFC, antral follicle count; AMH, anti-Müllerian hormone; CI, confidence interval; FSH, follicle stimulating hormone; GnRH, gonadotropin-releasing hormone; ICSI, intra-cytoplasmic sperm injection; IVF, in vitro fertilization; OR, odds ratio; PPOS, progestin-primed ovarian stimulation.

**Supplementary Table 2.** Results of the stratified analysis of the two COS regimens: GnRH antagonist (reference) and PPOS

| Parameters | Number of cycles | Cumulative live birth rate per oocyte retrieval cycle | |
| --- | --- | --- | --- |
|  |  | OR (95%CI) | *P*-value |
| Age, years |  |  |  |
| <35 | 463 | 0.56 (0.35, 0.88) | 0.0126 |
| ≥35 | 457 | 0.78 (0.46, 1.32) | 0.3595 |
| Insemination method |  |  |  |
| IVF | 656 | 0.74 (0.50, 1.09) | 0.1304 |
| ICSI | 245 | 0.37 (0.17, 0.80) | 0.0115 |
| Body mass index, kg/m^2^ |  |  |  |
| T1 (15.20–20.32) | 294 | 0.75 (0.41, 1.34) | 0.3267 |
| T2 (20.40–22.00) | 311 | 0.63 (0.34, 1.18) | 0.1498 |
| T3 (22.10–24.98) | 315 | 0.64 (0.35, 1.18) | 0.1514 |
| Type of infertility |  |  |  |
| Secondary infertility | 571 | 0.66 (0.43, 1.03) | 0.0673 |
| Primary infertility | 349 | 0.62 (0.35, 1.10) | 0.1051 |
| Duration of infertility, years |  |  |  |
| T1 (<2) | 216 | 0.44 (0.21, 0.92) | 0.0288 |
| T2 (≥2, <5) | 378 | 0.90 (0.52, 1.57) | 0.7179 |
| T3 (>5) | 325 | 0.55 (0.30, 1.01) | 0.0554 |
| Baseline FSH, U/L |  |  |  |
| T1（1.84–7.08） | 251 | 0.62 (0.35, 1.10) | 0.1027 |
| T2（7.09–9.67） | 253 | 0.68 (0.37, 1.25) | 0.2114 |
| T3（9.70–29.43） | 252 | 0.68 (0.35, 1.33) | 0.2565 |
| AMH, ng/mL |  |  |  |
| <1.2 | 419 | 0.62 (0.38, 1.03) | 0.0641 |
| ≥1.2 | 361 | 0.69 (0.43, 1.10) | 0.1178 |
| AFC, n |  |  |  |
| <5 | 345 | 0.73 (0.41, 1.30) | 0.2809 |
| ≥5 | 539 | 0.63 (0.41, 0.97) | 0.0344 |

When calculating the influence of regimen (GnRH antagonist or PPOS) on pregnancy outcome in a certain stratification, factors other than the stratification variables were adjusted. In the interaction test, age, body mass index, years of infertility, baseline FSH, AMH, and AFC were treated as continuous variables.

AFC, antral follicle count; AMH, anti-Müllerian hormone; CI, confidence interval; COS, controlled ovarian stimulation; FSH, follicle stimulating hormone; GnRH, gonadotropin-releasing hormone; ICSI, intra-cytoplasmic sperm injection; IVF, in vitro fertilization; OR, odds ratio; PPOS, progestin-primed ovarian stimulation

## Supplementary Figure

4110 cycles using PPOS or GnRH antagonist COS
January 2016 to December 2018

920 cycles included

1041 IVF/ICSI cycles

1. Non-POSEIDON population (n=1366)

2. Age >40 years, body mass index ≥25 kg/m^2^ (n=1703)

1. Chromosomal disease in either spouse (n=3)

2. Line PGT (n=10)

3. Uterine deformities (n=4)

Not possible to determine whether a cumulative live birth had been reached (embryos remaining) in n=104 cycles (PPOS group, n=71; GnRH antagonist group, n=33)

1024 IVF/ICSI cycles

**Supplementary Figure 1.** Flow chart of the data screening process.
COS, controlled ovarian stimulation; GnRH, gonadotropin-releasing hormone; ICSI, intra-cytoplasmic sperm injection; IVF, in vitro fertilization; PGT, preimplantation genetic testing; PPOS, progestin-primed ovarian stimulation.
